# Postharvest Storage Differentially Modulates the Enzymatic and Non-Enzymatic Antioxidant System of the Exocarp and Mesocarp of Hass Avocado: Implications for Disorders

**DOI:** 10.3390/plants12234008

**Published:** 2023-11-29

**Authors:** Rosana Chirinos, Jahaira Delgado-Pariona, Ana Aguilar-Galvez, Andrés Figueroa-Merma, Alejandro Pacheco-Ávalos, David Campos, Romina Pedreschi

**Affiliations:** 1 Instituto de Biotecnología, Universidad Nacional Agraria La Molina (UNALM), Av. La Molina s/n, La Molina, Lima 12056, Peru; dalexandradelgado@gmail.com (J.D.-P.); aaguilar@lamolina.edu.pe (A.A.-G.); 20131306@lamolina.edu.pe (A.F.-M.); 2 Programa de Investigación en Frutales, Universidad Nacional Agraria la Molina (UNALM), Av. La Molina s/n, La Molina, Lima 12056, Peru; aapachec@lamolina.edu.pe; 3 Escuela de Agronomía, Pontificia Universidad Católica de Valparaíso (PUCV), Calle San Francisco s/n, La Palma, Quillota 2260000, Chile; 4 Millennium Institute Center for Genome Regulation (CRG), Santiago 7800003, Chile

**Keywords:** *Persea americana*, postharvest storage, physiological disorders, antioxidant enzymes, phenolic compounds

## Abstract

The present study evaluated the performance of some enzymatic and non-enzymatic antioxidant systems against oxidative stress for 10 to 30 d of refrigeration (R) and 15 to 50 d in controlled atmosphere (CA) conditions in both exocarp and mesocarp of Hass avocados from early and late harvests and at shelf life (SL) or consumption maturity. The possible relationship of the antioxidant systems with the occurrence of physiological disorders is also evaluated. The results indicate that the enzymatic system—superoxide dismutase (SOD), peroxidase (POD), catalase (CAT), phenylalanine ammonium lyase (PAL) and polyphenoloxidase (PPO)—as well as the non-enzymatic system—such as phenolic compounds (PC)—showed different responses to the stress generated during storage and shelf life. In general, SOD, CAT, PAL and PPO did not significantly vary in storage (R or CA). At consumption maturity, SOD, POD and PAL activities decreased in the mesocarp (RSL and CASL), while CAT increased in the exocarp for CASL15-50d. PC instead decreased in the exocarp as the harvest period progressed while it increased in the mesocarp. Physiological disorders (dark spots) showed only in refrigeration on the exocarp at R30d and in mesocarp at RSL30d coincident with low SOD and low SOD and POD activity values, as well as low PC contents (*p*-coumaric and its derivatives and caffeic acid derivatives), respectively. The results support the use of CA as a postharvest technology to prevent the development of physiological disorders through the joint action of antioxidative defenses during avocado transport to distant markets until consumption maturity is reached.

## 1. Introduction

Avocado (*Persea americana*) var Hass is the most internationally traded cultivar, supported by its greater resistance to transport and handling conditions (as it has a hard peel), its easy recognition when it reaches its consumption maturity stage, evidenced by the change in skin color from green to dark or dark-violet, in addition to its appreciated sensory, nutritional and functional characteristics by the consumers [1]. The mesocarp, the most important edible portion of avocado, has a high content of monounsaturated fatty acids ~60–80% (especially oleic acid) [2], in addition to various bioactive compounds, including carotenoids, phytosterols, tocopherols, phenolic compounds, and others, that are recognized promoters of beneficial health effects based on their antioxidant properties [3,4].

Agroexports represent the driving force behind the economic and commercial development of the countries, generating opportunities for sustainable growth. In 2022, Peru and Chile led South American exports of Hass avocado, with USA and Europe as the main destinations [5]. The transport of avocado fruit from the place of production to distant markets (30–50 d) is a challenge for the operators involved in this activity. It includes the use of appropriate storage systems (e.g., including parameters) considering the long journeys to destinations (20–60 d) for sea shipping [6,7]. In addition, the fruit must fulfill the overall quality required by the final consumer.

Avocado is a climacteric fruit characterized by a high respiration rate and ethylene production during the ripening process [8]. Thus, to extend its shelf life and maintain its overall quality for exports, low-temperature storage becomes crucial. But, during storage at low temperatures, the fruit is susceptible to physiological disorders such as chilling injury, especially when stored for long periods and at temperatures below 3 °C [9], limiting the target markets. In view of this, another complementary postharvest technology known as controlled atmosphere (CA) is preferred, which, in addition to the use of low temperatures (4–7 °C), controls the concentration of gases (O_2_ and CO_2_) around the fruit [10]. CA not only extends the overall shelf life but reduces chilling injury and physiological and pathological disorders [7,11].

Avocado quality at the final destination is still a concern since it is reported that about 20% of exported avocados suffer various physiological disorders as a result of prolonged storage under cold and/or controlled atmosphere [12], internal defects such as darkening of the flesh (grayish flesh and vascular browning) [13] and external defects due to the presence of irregular black spots (lenticel damage, black spots, others) [14]. These disorders would also depend not only on the type and conditions of storage chosen but would also involve aspects related to the pre-harvest history of the fruit (i.e., drought, plant nutrition, temperature extremes, salinity and light) [15,16,17,18]. If physiological disorders are manifested at the “ready to eat” stage, the results are of great economic and image damage for the exporter country. Fruits with these characteristics are not marketed and/or purchased by consumers since the appearance of the fruit (external and internal) is used as a quality parameter to make the purchase at the moment [13] and, more importantly, the decision for future purchases.

Additionally, studies indicate that when fruits are at low storage temperatures, stress occurs, leading to the accumulation of a large amount of reactive oxygen species (ROS) in the cells [19], triggering oxidative stress. ROS are key regulators of a variety of processes including metabolism, growth and development, response to abiotic and biotic stresses, solute transport, autophagy and programmed cell death [20]. Different stresses can interact in complex ways to lead to a wide variety of metabolic responses, including both facultative metabolic adaptations that may afford stress protection, and metabolic impairments or injuries (e.g., inhibition of protein synthesis and membrane damage) [21] causing symptoms such as those of chilling injury disorder [22]. To combat oxidative stress, fruits deploy in response to their antioxidant defense system composed of the enzymatic system including superoxide dismutase (SOD), catalase (CAT), guaiacol peroxidase (POD), glutathione reductase, and others, and the non-enzymatic such as phenolic compounds, vitamin C, and flavonoids, among others [23]. These antioxidant defense systems play a role not only in maintaining ROS production, but also in determining the level of oxidative stress in avocado fruits [24]. Phenolic compounds as secondary metabolites can be synthesized in response to different situations and are antioxidants [25]. Phenolic compounds have been regarded to affect fruit quality characteristics and phenylalanine ammonia lyase (PAL) is a key enzyme involved in its biosynthesis. On the other hand, the participation of polyphenoloxidase (PPO) is associated with the development of browning, which is very evident at the mesocarp level of avocados during cold storage [26]. It has been reported that a large incidence of lenticel damage and lower activity of SOD, CAT, POD, PAL, phenolics and epicatechin levels may contribute to blackspot development in the exocarp during cold storage of Hass avocados [12,27].

The main objective of this research was to elucidate the changes established at the level of the antioxidant defense system of the exocarp (peel) and mesocarp (pulp) of Hass avocado stored under refrigerated (R) and controlled atmosphere (CA) conditions for a prolonged time. Thus, it included the evaluation of the enzymatic and non-enzymatic antioxidant defense system of the exocarp and mesocarp of Hass avocados from two harvests (early and late) each subjected to two storage conditions: refrigeration (7 °C, 85% RH) and controlled atmosphere (4% O_2_, 6% CO_2_, at 7 °C and 85% RH) and at their respective shelf life or consumption maturity (SL, 20 °C, 70% RH), and their possible relationship with the occurrence of physiological disorders.

## 2. Results

### 2.1. Physiological Disorders at the Exocarp and Mesocarp Level in Hass Avocados Stored in Refrigeration and Controlled Atmosphere and at Their Respective Consumption Maturity

**Figure 1:**
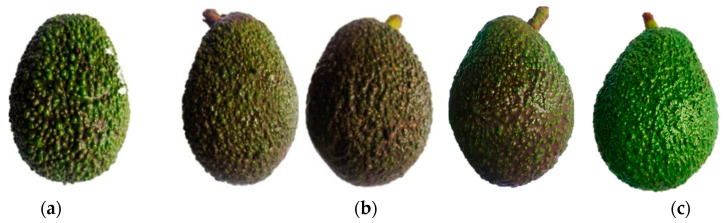
Dark spots on the exocarp of avocados var Hass at the exit of 30-day refrigerated storage corresponding to the early (**a**) and late (**b**) harvests. In (**c**) exocarp of avocado at the exit of refrigeration storage without any dark spot.

**Figure 2:**
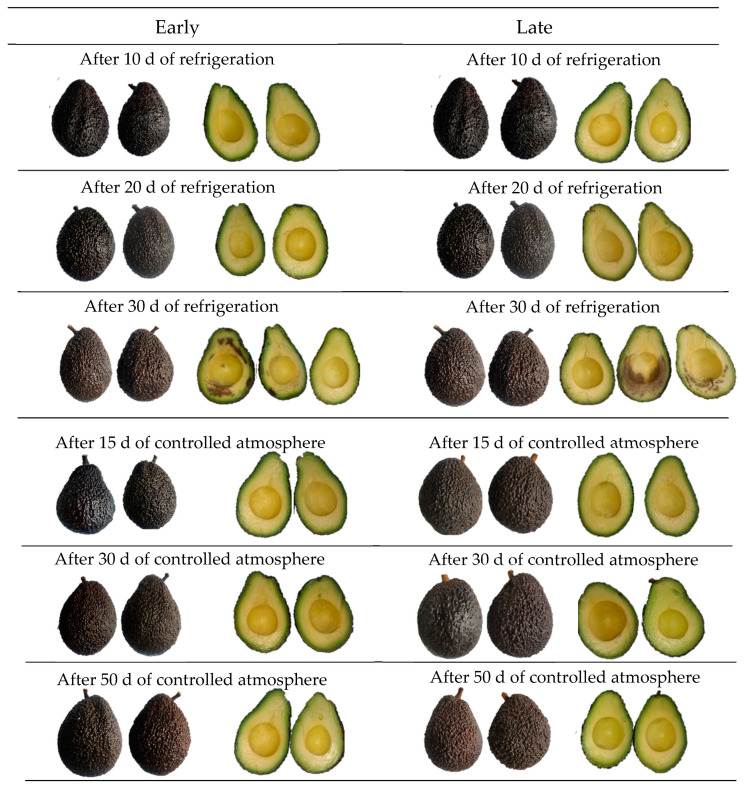
Images of exocarp and mesocarp of Hass avocados taken at consumption maturity or shelf life (SL, ~20 °C) of fruit previously stored under refrigeration for 10, 20 and 30 d and controlled atmosphere for 15, 30 and 50 d.

At the end of storage, it was observed that only the avocados that were refrigerated for 30 d (R30d) developed physiological disorders evidenced by the appearance of dark areas at the exocarp level. This incidence was lower in early harvest (1 of 20 fruits) compared to late harvest (4 of 20 fruits). Disorders were categorized according to the Agroforum scale (Supplementary Figure S1), as type 3 and 3–4 defects, respectively (Figure 1). Lenticel damage and dark spots of varying extents were evidenced. No defects were observed at the mesocarp level for all treatments evaluated, including R30d. Figure 2 shows the photographic records associated with the presence of physiological disorders in the exocarp and mesocarp of avocados after reaching consumption maturity or the ready-to-eat stage. The characteristic dark color developed in the exocarp in all treatments, even in those where surface damage was found (R30d). Instead, in the mesocarp of some fruits (R30d), pulp and vascular browning damages were observed, more evident and extensive in the late harvest (5 out of 10 fruits with defects type 4) compared to the early harvest (3 out of 10 fruits with defects type 3) fruit. Avocados under a controlled atmosphere did not present any damage.

### 2.2. Enzymatic and Non-Enzymatic Antioxidant System in Hass Avocados at Harvest, during Refrigerated and Controlled Atmosphere Storage and at Consumption Maturity

**Figure 3:**
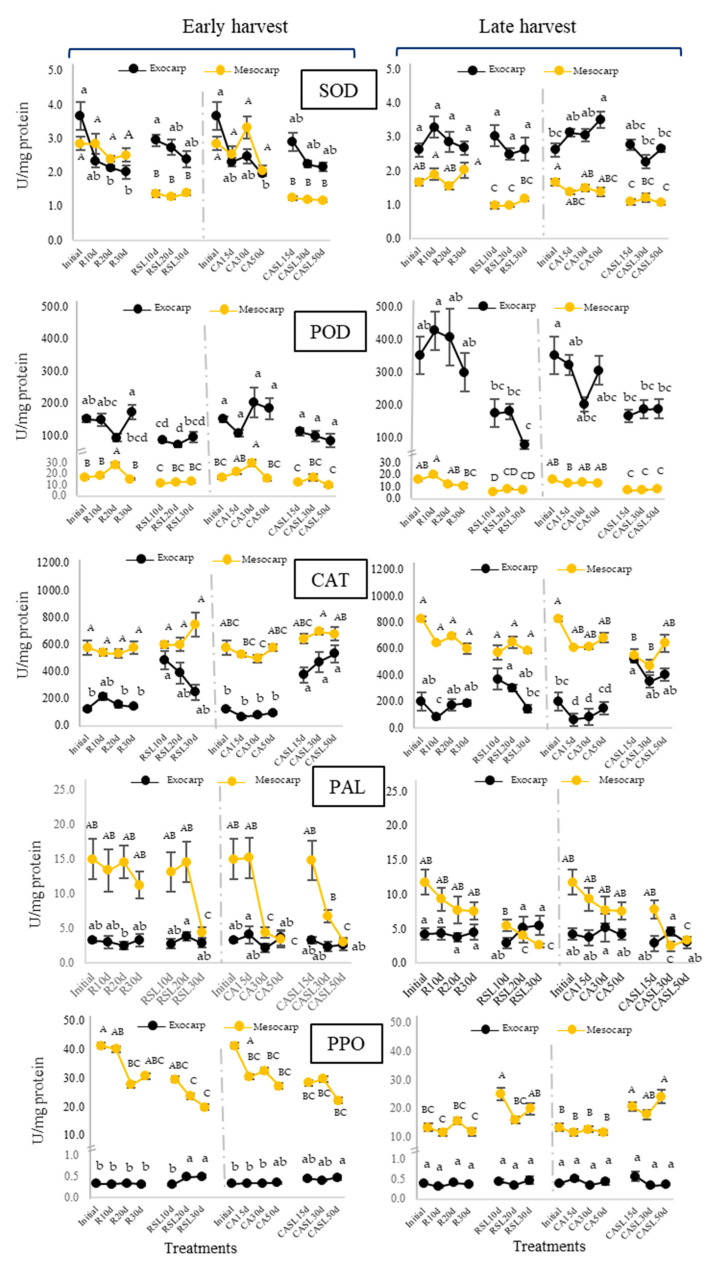
Changes in the enzymatic activities of superoxide dismutase (SOD), peroxidase (POD), catalase (CAT), phenylalanine ammoniolyase (PAL) and polyphenoloxidase (PPO) in the exocarp (black line) and mesocarp (yellow line) of Hass avocados stored under refrigeration for 10, 20 and 30 d (R10d, R20d and R30d) and controlled atmosphere for 15, 30 and 50 d (CA15 d, 30 d and 50 d) and at their respective consumption maturities (SL). Results are expressed as the mean (n = 10) ± standard error. Different capital letters and lowercase letters represent significant differences (*p* < 0.05) between mesocarp and exocarp samples, respectively, evaluated by storage type.

The changes in the enzymatic antioxidant system (SOD, POD and CAT enzymatic activities) in fruits stored in refrigerated (R) and controlled atmosphere (CA) conditions, in exocarp and mesocarp for the two harvests, are presented in Figure 3. SOD activity presented higher values in the exocarp than in the mesocarp, except for those found for the early harvest stored in R and CA, with very close values for both tissues. Exocarp SOD activity showed a decreasing trend during refrigeration (from R10d to 30d) but was not significant (*p* > 0.05) and there were no significant changes (*p* > 0.05) between storage exit time and consumption maturity (SL). Instead, mesocarp SOD activity displayed a significant decrease (*p* < 0.05) at consumption maturity, except in late-harvest avocados subjected to CA where no differences were found (*p* > 0.05). Contrary to SOD, higher values of POD enzyme activity were detected in the exocarp of late harvest (initial) compared to early harvest, with no differences at the mesocarp level.

In early harvest avocados, POD underwent modifications during storage in R, in the exocarp and mesocarp, some of them significant (*p* < 0.05). Instead, in CA, modifications of POD were observed only in the mesocarp of early harvest. On the other hand, in the exocarp, a tendency to decrease was observed from the moment of the end of storage until the arrival of consumption maturity (SL), being this decrease significant (*p* < 0.05) only in the samples kept for 30 d in refrigeration (RSL30d) for the two harvests evaluated. A tendency to decrease was observed also in the mesocarp. CAT activity in the exocarp and mesocarp had higher values in late harvest (initial) than early harvest (initial) fruit. For the most part, mesocarp CAT activity did not show significant differences (*p* > 0.05) between different storage times and at consumption maturity. With respect to the exocarp, CAT values tended to increase significantly (*p* < 0.05) at consumption maturity under CA (CASL), not showing the same trend under refrigerated conditions.

In addition to the enzymes SOD, POD and CAT, known for their antioxidant performance, the participation of other enzymes such as PAL and PPO was evaluated (Figure 3). Regarding PAL activity in the exocarp, no significant changes (*p* > 0.05) were observed in all the samples evaluated. A decreasing trend for PAL in the mesocarp of Hass avocados stored either in R or CA was observed when consumption maturity was reached, which was significant (*p* < 0.05) for RSL30d and CASL30-50d in early and late harvests. PPO activity was expressed to a greater extent in the mesocarp with respect to the exocarp, the latter tending not to undergo major changes in all the samples analyzed. PPO activity in the mesocarp of early harvest avocados tended to decrease during storage (in R and CA) continuing this behavior until the end of their shelf life (RSL and CASL). Instead, late-harvest avocados displayed no significant changes (*p* > 0.05) during storage, but there was a tendency to increase once consumption maturity was reached.

**Figure 4:**
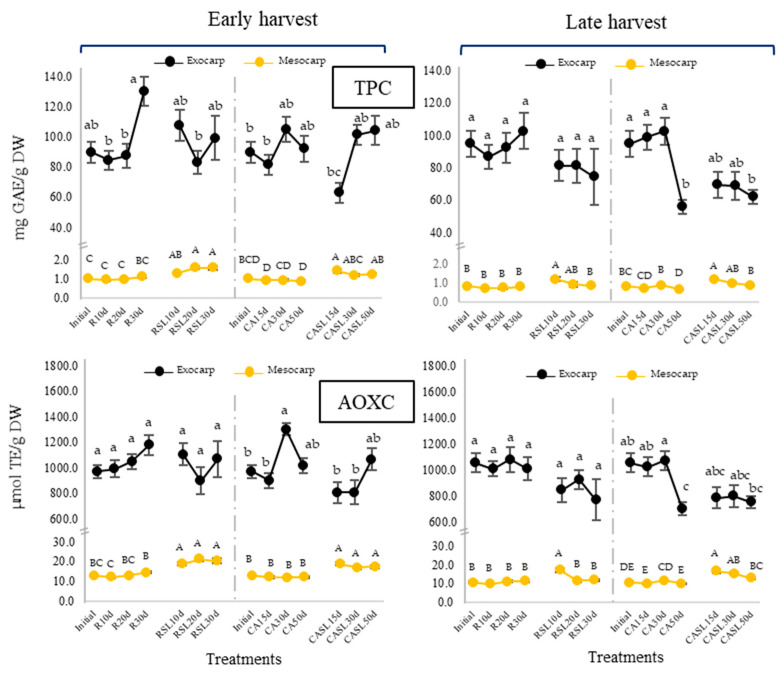
Changes in total phenolic compound content (TPC) and antioxidant capacity (AOXC) in the exocarp (black line) and mesocarp (yellow line) of Hass avocados stored using refrigeration for 10, 20 and 30 d (R10d, R20d and R30d) and controlled atmosphere for 15, 30 and 50 d (CA15 d, 30 d and 50 d) and at their respective consumption maturities (SL). Results are expressed as the mean (n = 10) ± standard error. Different capital letters and lowercase letters represent significant differences (*p* < 0.05) between mesocarp and exocarp samples, respectively, evaluated by storage type.

The non-enzymatic antioxidant system represented by total phenolic compounds (TPC) and AOXC displayed notably higher values in the exocarp compared to the mesocarp (Figure 4). A significant increase in TPC (*p* < 0.05) was observed for exocarp samples corresponding to R30d of early harvest but a significant decrease for CA50d of late harvest. Non-notorious changes were observed at the mesocarp level except for CASL15-50d of late harvest, with most of the results related to the AOXC trends. Moreover, TPC and AOXC showed a tendency to increase at consumption maturity in the mesocarp, except for RSL20d-30d of late harvest.

### 2.3. Profile of Phenolic Compounds Determined by UPLC-PAD in Hass Avocados at Harvest and at Consumption Maturity Subjected to Different Storage Treatments

The profile of total phenolic compounds and their participation in quantity was evaluated in freshly harvested avocados (initial) and at their respective consumption maturity for both harvests (Table 1 and Table 2). Chlorogenic acid and epicatechin were identified in the exocarp of avocados, as well as a greater number of derivatives of the flavanol family with absorption spectra similar to those of epicatechin, and thus identified as epicatechin derivatives 1–5 (Table 1).

The content of total phenolic compounds (TPC-UPLC) decreased in quantity from early (I) to late harvest (II), also at late harvest some phenolics found in early harvest were not detected (Table 1). Twenty-day refrigerated ripe avocados (RSL20d) from early harvest showed higher TPC-UPLC contents compared to the other storage times (RSL10d and RSL30d). Similar behavior was displayed by CA samples (CASL20d > CASL10d and 30d). Late-harvest fruit displayed a significant increase in TPC-UPLC from RSL10d to RSL 30d, while in the CASL15d to CASL30d treatments, it remained unchanged.

Regarding the profile and content of phenolic compounds identified in the mesocarp (Table 2), only *p*-coumaric acid was identified; the other compounds corresponded mostly to phenolics of a similar spectrum to hydroxycinnamic acids of the *p*-coumaric type, followed by caffeic acid and hydroxybenzoic acid of the syringic type. In freshly harvested avocados for both harvests (I and II), fewer phenolic compounds were detected (four), which could not be quantified due to their low concentration. A significant increase in phenolic compounds of the hydroxycinnamic acid type was observed in late harvest for all the samples evaluated at consumption maturity; on the contrary, the syringic acid derivative decreased. TPC-UPLC for all samples was significantly higher (*p* < 0.05) in quantity in the late harvest supported by the higher participation of hydroxycinnamic acids. The final TPC-UPLC content in the mesocarp for cold-stored avocados RSL (10 and 20 d) and controlled atmosphere CASL (15 and 30 d) was similar (*p* > 0.05) in both harvests. The lowest values (*p* < 0.05) of TPC-UPLC were found in RSL30d samples, followed by CASL50d.

### 2.4. Multivariate Analysis of the Variables Evaluated in Hass Avocado at Harvest and Consumption Maturity

**Figure 5:**
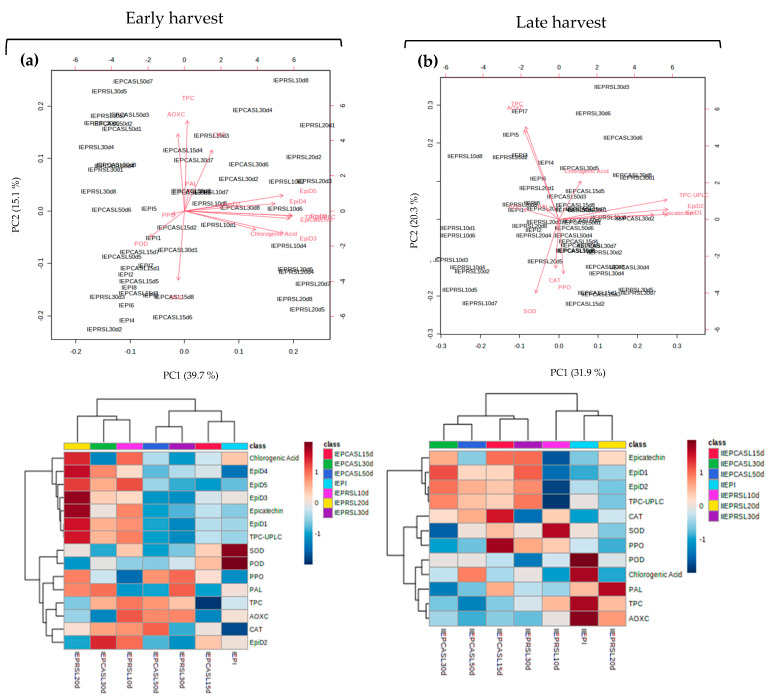
Biplot and heat maps for exocarp (EP) displaying the samples and variables overlaid for the early (**a**) and late-harvest (**b**) datasets. IEPI and IIEPI = initial samples at early (I) and late (II) harvests. R10d, R20d and R30d = avocados cold stored for 10, 20 and 30 d. RSL10d, RSL20d and RSL30d = at consumption maturity from refrigerated samples for 10, 20 and 30 d. CA15d, CA30d and CA50d = avocados stored for 15, 30 and 50 d under controlled atmosphere. CASL15d, CASL30d and CASL30d = at consumption maturity from controlled atmosphere for 10, 20 and 30 d. SOD = superoxide dismutase, POD = peroxidase, CAT = catalase, PAL = phenylalanine ammoniolyase, PPO = polyphenoloxidase, TPC = total phenolic compounds, AOXC = antioxidant capacity, TPC-UPLC = total phenolic compounds quantified by UPLC, EpiD1-D5 = epicatechin-derived phenolic compounds 1 to 5.

**Figure 6:**
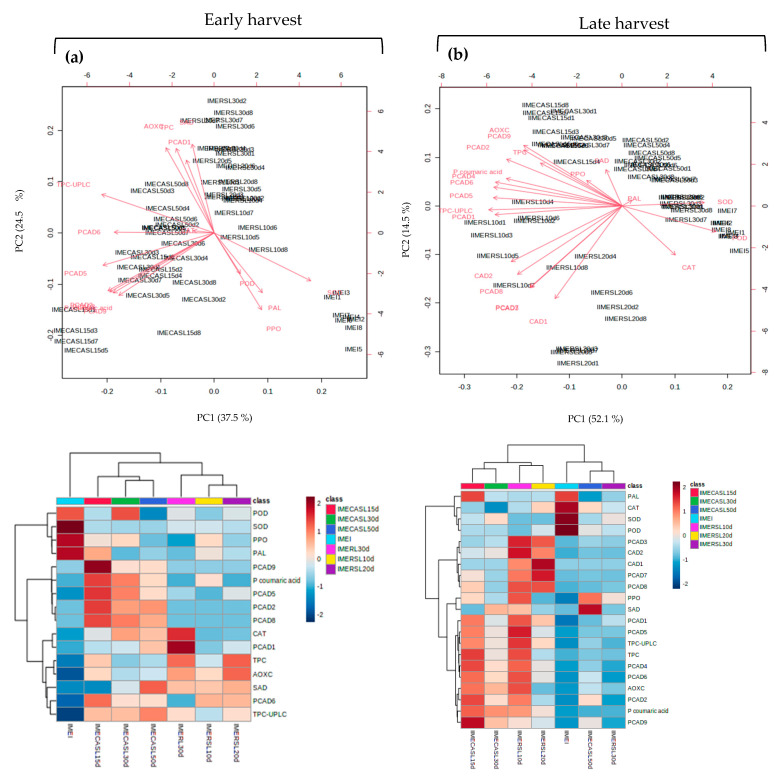
Biplot and heat maps for mesocarp (ME) displaying the samples and variables overlaid for the early (**a**) and late harvest (**b**) datasets. IMEI and IIMEI = initial samples at early (I) and late (II) harvests. R10d, R20d and R30d = avocados cold stored for 10, 20 and 30 d. RSL10d, RSL20d and RSL30d = at consumption maturity from refrigeration for 10, 20 and 30 d. CA15d, CA30d and CA50d = stored for 15, 30 and 50 d under controlled atmosphere. CASL15d, CASL30d and CASL30d = at consumption maturity from controlled atmosphere for 10, 20 and 30 d. SOD = superoxide dismutase, POD = peroxidase, CAT = catalase, PAL = phenylalanine ammoniolyase, PPO = polyphenoloxidase, TPC = total phenolic compounds, AOXC = antioxidant capacity, TPC-UPLC = total phenolic compounds quantified by UPLC, PCAD1-D8 = *p* coumaric acid-derived phenolic compounds 1 to 8, CAD1-2 = caffeic acid-derived phenolic compounds 1 and 2, SAD = syringic acid-derived phenolic compound.

Figure 5 and Figure 6 show the biplots and heatmaps of the samples and overlapping response variables obtained for the exocarp (EP) and mesocarp (ME) of early (a) and late (b) harvests, respectively. The corresponding score plots are presented in Supplementary Figure S2. Principal component analysis (PCA) explained a total of 54.8 and 52.2% of the variance with the first two components (PC1 and PC2) for the exocarp of early and late harvest fruit (Figure 5). Instead, PCA analysis for the mesocarp of early and late harvest fruit (Figure 6) explained with the two first components up to 62.0% and 66.6% of the variance.

Early harvest fruit (IEP, Figure 5a) exocarp presented high values of SOD and POD in freshly harvested avocados (IEPI) followed by CASL15d. The highest values for CAT were associated with CASL50d, CASL30d and RSL10d. Epicatechin-type phenolic compounds and their derivatives were found in high amounts in RSL20d, RSL10d and CASL30d. RSL30d and CASL50d had in common low contents of epicatechin-type phenolic compounds and their derivatives. Instead for late harvest (IIEP, Figure 5b), high SOD, POD and CAT activities were associated with RSL10d, EPI (initial) and CASL15d, respectively. Phenolic compounds of the epicatechin type and their derivatives were found in higher amounts in the CASL15-50d and RSL30d treatments.

Early-harvest (MEI, Figure 6a) mesocarp presented high POD values in freshly harvested avocados (MEI) and CASL30d. Instead, higher values of CAT occurred at RSL30d and for POD at MEI. Hydroxycinnamic acids of the *p*-coumaric type were mostly found in higher amounts in the CASL15-50d treatments, while the syringic acid derivative was present in higher amounts in the RSL10-30d and CASL50d treatments. Results for late harvest (MEII, Figure 6b) matched the early harvest with high values of SOD, POD, CAT and PAL in the freshly harvested samples (MEI), higher values of *p*-coumaric acid derivatives in samples from CASL15d and RSL10d, followed by CASL30d and RSL20d. At both early and late harvests, the RSL30d treatment displayed the lowest contents of phenolic compounds of the *p*-coumaric type and its derivatives and caffeic acid derivatives, as well as the lowest SOD and PAL activities.

## 3. Discussion

### 3.1. Physiological Disorders at the Level of Exocarp and Mesocarp

Chilling injury symptoms in avocados have been attributed to several factors including low-temperature storage, high concentration of ethylene, the harvest of over-ripened fruits and the specific conditions of each producing region [18]. It is known that avocado is a cold-sensitive fruit, so if storage temperatures are not properly managed, physiological disorders may occur in both pulp and peel [28]. Thus, it is advisable to apply storage temperatures between 6 to 8 °C for 2 to 4 weeks to prolong its shelf life [9]. After 4 weeks, pitting and blackening of the peel and browning and darkening of the vascular filaments in the pulp may occur [29]. Other research has shown that physiological disorders appear under regular air storage at temperatures close to or below 3 °C, while at temperatures between 4 and 7 °C, the rate of ethylene synthesis and the respiratory rate of the fruit are reduced, thus decreasing its metabolism, and increasing the potential storage period [9,13,30]. In this study, the presence of damage in the exocarp of avocados at the exit of cold storage (7 °C) for 30 d (R30d) in early harvest and with greater presence in late harvest (without any incidence in the mesocarp), was associated with the development of lenticel damage and dark spots. Previous studies have detected black spots in the exocarp of avocado var Hass after 40 days of cold storage at 5 °C [17]. Likewise, the presence of lenticel damage and black spots was detected in early and middle harvest fruit at the exit of storage under CA at 5 °C (4% O_2_ and 6% CO_2_) for 55 days and regular air at 5 °C for 30 days, as it is more present in regular air storage and middle-harvest avocados [27]. The appearance of dark spots on the exocarp has been associated with low dry matter content [17]. In our study, the lowest dry matter values were found in the exocarp of late harvest (20.0–23.7%) compared to early harvest (24.6–25.3%) and it was at late harvest where the greatest development of physiological disorders occurred (R30d). High dry matter contents are related to higher participation of compounds in the exocarp, including those of an antioxidant nature, which may have contributed to the control of oxidative damage in early-harvest fruits as opposed to late harvest.

On the other hand, Sharma et al. [31] reported that chilling injury symptoms become more pronounced in the flesh when the fruit is transferred from cold storage to ambient, shelf-stable conditions for marketing. Damage at the mesocarp level in the RSL30d treatments was related to the physiological disorder present in the exocarp at the exit of refrigeration (R30d), as it is more pronounced in late-harvest fruit. The prolonged cold storage times (R), together with the changes associated with the integrity of the cellular tissue in the mesocarp, related to the advance of fruit senescence under shelf-life conditions, would have triggered a series of oxidative reactions with the consequent formation of dark spots in the pulp and vascular browning of varying extent, where the antioxidant defense systems, both enzymatic and non-enzymatic, could not control the oxidative stress established. The above-mentioned defects in the exocarp and mesocarp were not observed in avocados stored in CA, even for much longer storage times (50 d) than under refrigeration. Under cold conditions and with a composition of the atmosphere surrounding the fruit composed of low levels of oxygen and higher levels of carbon dioxide, different from regular air, the integrity of the cell wall of the fruit is maintained by reducing the activity of the enzymes that degrade them, as well as the activity of the oxidative enzymes [31,32]. In addition, the activation of anabolic and catabolic pathways might contribute to the antioxidant status of the fruit. For the Hass variety, it is recommended to store them in CA at a concentration of O_2_ (2–10%) and CO_2_ (4–10%), for a period not exceeding 9 weeks and then at 20 °C to reach consumption maturity with an optimum quality [29]. External pre-harvest factors that could contribute to the appearance of stress such as the origin, fruit size, fruit composition and crop nutrition [16,31,33] could be added to the results found for the postharvest treatments studied [16,31,33].

### 3.2. Enzymatic and Non-Enzymatic Antioxidant System in Hass Avocados at Harvest, during Storage under Refrigeration and Controlled Atmosphere for Prolonged Periods and at Their Consumption Maturity

During storage conditions and when the avocado reached consumption maturity, oxidative stress was present to a greater or lesser extent depending on the conditions to which the avocado was exposed and the response of the endogenous antioxidant system of the fruit. The antioxidant enzymes SOD, POD and CAT showed different responses depending on the tissue evaluated, since there were enzymes with a more active role in the exocarp and others in the mesocarp. The higher activity of SOD in the exocarp compared to the mesocarp indicates that its participation is important in this tissue to counteract the stressful conditions to which the fruit was exposed as a first defense. Previous studies have reported that cold conditions did not affect the mode of action of SOD [34,35]. When the fruit reaches consumption maturity, there is a high oxidative metabolism, which involves the consumption of oxygen favoring the formation of superoxide radicals, this being dissmuted by SOD to H_2_O_2_ [36], a reaction that would have been present in shelf-life conditions (SL) at the exocarp level, favored by the high activity of this enzyme. On the contrary, in the mesocarp, the significant decrease in SOD at SL conditions could be due to its intense participation to protect the mesocarp, generating and accumulating high amounts of H_2_O_2_ among other derived products, which would have participated as inhibitors limiting its action [37,38]. H_2_O_2_ is an oxygen reactive species that can be degraded by POD and CAT enzymes. POD participates in the elimination of excess reactive oxygen species, such as H_2_O_2_, and is also capable of reducing the generation of free radicals and neutralizing the excess of free radicals created by stress conditions [39]. Therefore, SOD and POD would act complementing their activities under the stress conditions presented. As with SOD, POD exhibited higher activity in the exocarp, but its activity decreased in the exocarp and mesocarp when the avocados ripened after storage, a result previously found in the mesocarp [38,40]. It has been reported that POD is also able to catalyze the oxidation of phenolic compounds in the presence of H_2_O_2_ to form brown compounds [41]. This reaction could have contributed to the appearance of dark spots at the exocarp level in 30 d refrigerated avocados (R30d) and in the mesocarp of 30 d refrigerated avocados evidenced at consumption maturity (RSL30d). No evidence of damage was found under CA storage given the limited oxygen condition that prevents the oxidation process of the fruits, preventing the appearance of the dark spots. In contrast to SOD and POD, CAT was present in higher amounts in the mesocarp than in the exocarp, indicating that the action of this enzyme would be of great importance to stop the oxidative reactions in the mesocarp related to its mechanism of action. CAT catalyzes the dismutation of two H_2_O_2_ molecules into water and oxygen and is reported to be more effective in H_2_O_2_ degradation than POD, and CAT also tolerates high peroxide concentrations [42]. Therefore, in the exocarp and mesocarp, CAT would have actively participated in eliminating H_2_O_2_ produced by SOD and supporting POD, always showing an activity without significant changes. The exception was the notorious CAT increase in the exocarp of CASL15-50d avocados from both harvests, probably related to conditions that favor its activity increase (e.g., presence of substrates, concentration, pH, etc.).

Other enzyme activities tested were PAL and PPO. PAL is the enzyme responsible for the synthesis of polyphenols in plants and catalyzes the elimination of an ammonium converting phenylalanine into cinnamic acid, a precursor of simple phenols such as coumaric and benzoic acids, and complex phenols such as flavonoids and stilbenes, among others [43]. In general, this enzyme always maintained its phenolic synthesis activity at the exocarp and mesocarp levels during storage either in R or CA, indicative of not being greatly affected by both conditions. But, a notorious decrease was observed at the mesocarp level at consumption maturity, especially in the RSL30d and CASL30-50d treatments. PPO catalyzes two different reactions in the presence of O_2_: the *o*-hydroxylation of phenolic substrates to *o*-diphenols and the oxidation of *o*-diphenols to quinones in the presence of oxygen; these quinones can spontaneously polymerize through non-enzymatic routes generating brown pigments known as melanins [44]. PPO was found to be more active in the mesocarp than in the exocarp, possibly due to the availability of its substrates in this tissue. PPO activity has been associated with symptoms of chilling injury and high PPO activity together with reduced tissue integrity would lead to severe browning of the fruit [26]. PPO activity in the mesocarp of late-harvest avocados showed a tendency to increase. High PPO activity was also found in CASL avocados but was not related to browning incidence, probably due to fewer substrates or the presence of other antioxidant defenses that prevented the triggering of oxidative stress. Normally, PPO, oxygen and phenolic compounds are separated through a series of membrane systems. However, continuous stress from reactive oxygen species caused by prolonged chilling leads to cell membrane rupture, with consequent loss of cell compartmentalization, leading to the release of enzymes that, in contact with their substrates, trigger browning [43].

During storage, it was observed that TPC, quantified spectrophotometrically in the exocarp and mesocarp, varied little (except for early-harvest R30d and late-harvest CASL50d, where significant changes were evidenced). A higher amount of phenolics was observed, especially in the mesocarp at consumption maturity (SL), a net result of the synthesis, degradation and biotransformation of phenolic compounds that occurs during avocado ripening [45]. AOXC was related to TPC, so increases in AOXC at consumption maturity (SL) would be related to the synthesis of phenolic compounds in avocado during ripening: compounds such as chlorogenic acid, vanillic acid, protocatechuic acid, among others [45], which would improve the antioxidant capacity and the ability to eliminate reactive oxygen species [26].

### 3.3. Total Phenolic Compounds (UPLC-DA) in Hass Avocados at Harvest and at Consumption Maturity of Samples from Refrigeration and Controlled Atmosphere Treatments

To obtain more information related to phenolic compounds as an antioxidant system, a more refined evaluation was made with respect to the spectrophotometric method, using UPLC-PAD. The profile of the main phenolic compounds and their concentrations in the exocarp and mesocarp of avocado was determined by UPLC-PAD at harvest (initial) and at consumption maturity (RSL10-30d, CASL15-50d). In terms of participation and evolution of TPC-UPLC, it was observed that they were related to spectrophotometrically determined TPC. The UPLC-PAD results show differences in the type and content of phenolic compounds synthesized in the exocarp and mesocarp. In the exocarp, a significant presence of phenolics of the flavonoid family of the epicatechin type and a representative of the hydroxycinnamic acids, chlorogenic acid, was detected. Previous studies have reported hydroxybenzoic and hydroxycinnamic acid derivatives, free and glycosylated flavonoids, epicatechin derivatives, catechins and dimeric and trimeric procyanidins with different degrees of polymerization in avocado peel [45,46,47]. In the mesocarp, a higher component of hydroxycinnamic acids of the *p*-coumaric type and its derivatives was found, followed by derivatives of caffeic acid, as well as a derivative of syringic acid, which agrees with other research studies [38,48]. The high concentrations found by UPLC-PAD in the exocarp with respect to the mesocarp would justify the role of the exocarp as a tissue that protects the fruit from aggressive situations of the external environment, to which the fruit is exposed in pre- and postharvest, requiring the synthesis of antioxidant components to cope with potential biotic and abiotic stress processes. The exocarp of the avocado fruit plays an essential role in the regulation of gas and water exchange of the whole fruit, filtering damaging UV light, providing chemical support, preventing organ fusion and limiting invasion by pathogens [49]. The exocarp has a higher free radical scavenging capacity compared to the mesocarp and this is associated with the presence of a higher content of phenolics, as well as other bioactive compounds [50]. The decrease in the number of phenolics detected and their concentration between early and late harvests would account for their consumption in response to stress during their stay in the field prior to harvest, in addition to storage time and type. It was observed that antioxidant phenolics increased in refrigerated avocados, from the initial stage reaching a maximum at RSL20d, dropping dramatically at the RSL30d stage. Although the evolution of phenolics under CASL was like those developed under RSL, the modification in the composition of O_2_ and CO_2_ gases in the fruit environment would have helped to slow down the appearance and development of the damage present at the exocarp level for up to 50 d.

In relation to the evolution of phenolic compounds in the mesocarp, it was observed that, unlike what was found in the exocarp, under ready-to-eat conditions, regardless of the storage treatment, most of them were synthesized in late-harvest conditions and those that were present from the beginning increased in quantity. The appearance of a higher content of phenolic compounds would have been a consequence of the loss of the integrity of its protective layer (exocarp) due to oxidative stress, which, added to the prolonged storage time and the consequent senescence, induced the fruit to deploy its antioxidant defense system, including phenolic compounds, releasing them into the environment. During maturity and storage, extensive remodeling of the cell wall structure of the epidermis occurs and enzymes such as pectinases, cell wall expansins and non-enzymatic mechanisms have been implicated in epidermis degradation [51]. It is reported that phenolics in the mesocarp are mostly found in the bound form at the cellular matrix level and in smaller amounts as free, which would also influence a reduction in their ability to function as antioxidants and neutralize free radicals produced in this tissue [52], explaining this because the mesocarp has less content of phenolic compounds and AOXC compared to the exocarp. The increase in phenolics in the mesocarp would be due to the release of phenolics bound to their free forms to act as antioxidants.

At this point, evaluating all the results found and their possible relationship with the physiological disorder found in the avocados, first, it should be noted that this took place in avocados subjected to refrigerated storage for long periods of time (30 d). The browning (lenticel damage and dark spots) evidenced in the exocarp at R30d could be related to the low SOD activity and a higher incidence in late-harvest fruits with low exocarp dry matter contents. Our results are partially in agreement with Uarrota et al. [12] who reported that the large incidence of lenticel damage in avocados and lower activity of SOD, CAT, POD and PAL may contribute to black spot development during cold storage. In mesocarp of RSL30d samples, where pulp and vascular browning-type alterations were present, the disorder coincided with low content of phenolic compounds (*p*-coumaric and its derivatives and caffeic acid derivatives) as well as low SOD and PAL activities, which is more evident with the advancement of the harvest.

Finally, it is important to mention that the damages triggered by ROS are compensated in the fruit by the activation of different antioxidant defense enzymes, such as SOD, POD, CAT, glutathione reductase and also non-enzymatic including ascorbic acid, tocopherols, carotenoids, flavones, anthocyanins and glutathione (GSH), that play a role not only in maintaining ROS production, but also determining the level of oxidative stress in avocado fruits [24]. These antioxidant defenses could have participated simultaneously under the stress conditions presented in this study and would have impacted the results obtained.

## 4. Materials and Methods

### 4.1. Sampling Material

Avocado (*Persea americana*) var Hass, from the field of the Fruit Tree Research and Social Projection Program of the Universidad Nacional Agraria La Molina, was used. One hectare of the field was randomly sampled, from which a total of 150 fruits (per harvest) were harvested from 15–20 avocado trees. The early (I, mesocarp dry matter: 22.12 ± 1.44%) and late (II, mesocarp dry matter: 30.82 ± 1.74%) harvests were carried out in April and July 2021, respectively. The fruits were quickly transported to the laboratory, where they underwent a selection stage (removal of samples with signs of deterioration) and cleaning (with a squeegee). A total of 10 avocados were randomly selected per harvest to determine the initial characteristics of the fruit (Initial = 0 d), while the rest of the avocados were coded from 1 to 130, and then distributed according to the postharvest storage treatment to be evaluated.

### 4.2. Postharvest Storage: Refrigeration and Controlled Atmosphere and Shelf-Life Conditions

For refrigerated storage, three storage periods were used: 10, 20 and 30 d (R10d, R20d and R30d). The fruits were placed in plastic boxes and were covered with a perforated low-density polyethylene bag inside a cold room at 7 °C and 85–90% RH. For each storage time, a total of 20 units per box were available. At the end of the storage period, one box was removed from which 10 avocados were taken as samples for the respective period, and the remaining 10 avocados were placed in shelf-life conditions (SL) (20 °C and 70% RH) until they reached consumption maturity (firmness of 4–8 N) [53].

Controlled atmosphere storage was carried out in an atmosphere composed of 4% O_2_, 6% CO_2_ at 7 °C [38] for three time periods: 15, 30 and 50 d (CA15d, CA30d and CA50d). Three microchambers were used (with 20 fruits per chamber), each representing one storage period. Once the periods were completed, the fruits were removed, of which 10 represented the respective period, and the other 10 remaining fruits were placed in the shelf-life conditions (SL) until they reached consumption maturity, following the conditions described in the previous paragraph.

For all sampling: Initial, refrigerated storage (R10d, R20d and R30d) and at consumption maturity (RSL10d, RSL20d and RSL30d), controlled atmosphere storage (CA15d, CA30d and CA50d) and at consumption maturity (CASL15d, CASL30d and CASL50d), avocados were quickly split in half and photographed. The total of both components per avocado were pulverized under liquid nitrogen, stored in high-density polyethylene bags with hermetic seal and stored at −80 °C until analysis.

### 4.3. Dry Matter Content

Dry matter was measured in the exocarp and mesocarp tissues according to the percentage gravimetric method of the AOAC Method 920.151 [54]. Moisture was calculated and the dry matter (DW) content was obtained by difference with the 100 percent moisture content.

### 4.4. Assessment of Exocarp and Mesocarp Physiological Disorders

For this part, the presence or absence of dark spots on the exocarp of avocados corresponding to black spot or lenticel damage and the presence or absence of pulp and vascular browning in the mesocarp were evaluated from photographic records. For this purpose, the fruits per treatment with the presence of disorder were first identified and then the degree of damage was evaluated according to a qualitative–quantitative scale ranging from 1 to 5 according to Agroforum [55], where 1 = no damage and 5 = damage over a large surface area (Supplementary Figure S1).

### 4.5. Specific Enzymatic Activities

The activities of the enzymes superoxide dismutase (SOD), catalase (CAT), peroxidase (POD), polyphenol oxidase (PPO) and phenylalanine ammonia lyase (PAL) were conducted according to methodologies previously developed, well-established and reported in different publications [12,17,38]. A UV-Vis microplate reader (Biotek, VT, USA) was used for all measurements. The enzymatic extracts obtained from the above mentioned methodologies had their soluble protein content determined by the Bradford method using bovine serum albumin as standard [56], expressing the results as mg protein/mL. The enzyme activity value (U) of SOD corresponded to the amount of enzyme required to inhibit the nitro blue tetrazolium (NBT) by 50%. CAT activity value (U) corresponded to the mM hydrogen peroxide formed per min and POD and PFO activity values corresponded to the variation of absorbance over time from the slope of the linear section of the reaction curve. PAL activity (U) was determined by cinnamate production over time (min). For the calculation of the specific enzyme activity, the U value was divided by the soluble protein content, and the results were finally expressed as U/mg protein.

### 4.6. Determination of Total Phenolic Compounds and Antioxidant Capacity

Prior to the determination of the content of total phenolic compounds and antioxidant capacity, the antioxidant extracts were obtained. In the case of the exocarp, the extract was obtained using 70% (*v*/*v*) acetone as solvent under agitation (200 rpm) for 90 min [57], followed by centrifugation (4000× *g*, 15 min, 4 °C) and subsequent recovery of the supernatant. The mesocarp was subjected to extraction with 80% (*v*/*v*) methanol for 60 min [4] followed by centrifugation (4000× *g*, 15 min, 4 °C).

Total phenolic compounds (TPC) were quantified using the Folin-Ciocalteau method described by Singleton and Rossi [58]. Sample absorbance was measured at 755 nm and values were expressed as mg of gallic acid equivalents (GAE)/g of dry weight (DW). The antioxidant capacity (AOXC) was determined by the ABTS assay adapted from Arnao et al. [59]. The sample absorbances were measured at 734 nm, and the antioxidant capacities were calculated in µmol Trolox equivalents (TE)/g DW.

### 4.7. Profile and Content of Phenolic Compounds Determined by UPLC-PAD

The profile and content of phenolic compounds detected and identified by UPLC-PAD were carried out on samples corresponding to early and late harvests (initial) and on samples derived from all treatments only at consumption maturity (SL). For this evaluation, the extracts obtained were used for the determination of AOXC. Phenolic compounds were identified and quantified using the UPLC Acquity H-Class (Wa-ters) system coupled to a PDA detector (eʎ detector) and Empower II software (version year 2005-2008). An Acquity BEH C_18_ column (1.7 µm, 100 mm × 2.1 mm) (Waters) with a BEH C_18_ (1.7 µm, 5 mm × 2.1 mm) column guard was used. The mobile phase was composed of (A) 0.1% formic acid in MilliQ water and (B) acetonitrile with 0.1% formic acid. The gradient used was as follows: 2% B for 2 min, 2–7% B in 2 min, 7–12% B in 11 min, 12–26% B in 5 min, 26–55% B in 5 min, and 95% B for 3 min and the column was equilibrated with 2% B for 5 min. Samples were run at a flow of 0.2 mL/min at 30 °C. Phenolic compounds detected were identified and quantified by comparing their retention time and UV-visible spectral data (recorded from 200 at 700 nm) with a previously known injected standard at 280, 320 and 360 nm. The results were expressed in mg/100 g DW.

### 4.8. Statistical Analysis

The results were reported as mean ± standard deviation or standard error of ten independent biological replicates (ten independent fruit). Analysis of variance (ANOVA) was used to compare the means, and Tukey’s test was used to assess statistically significant differences among treatments (*p* < 0.05). All statistical analyses were performed with Statgraphics Centurion 19-X64 (Stat Point Technologies, Inc., Warrenton, VA, USA). Principal component analysis (PCA) and hierarchical clustering analysis (using Euclidean distance and the Ward algorithm) were performed on the normalized data using MetaboAnalyst 5.0 (Xia Lab, McGill University, Montreal, QC, Canada).

## 5. Conclusions

The results indicate that the enzymatic and non-enzymatic antioxidant defense systems were able to cope with the oxidative stress caused by the storage conditions to which the Hass avocado was exposed as well as by the advance of its senescence under shelf-life conditions. The presence and response of the antioxidant systems evaluated varied according to the avocado tissue studied. Thus, SOD and POD were present with greater activity in the exocarp, while in the mesocarp it was CAT, as well as the PAL and PPO enzymes. In general, under R and CA storage, SOD, CAT, PAL and PPO enzymes, both at the exocarp and mesocarp levels, did not significantly change their activities. But, some of them presented a decreasing activity trend as storage time increased. At consumption maturity (SL), different behaviors were observed. For instance, SOD, POD and PAL activities decreased in the mesocarp, independent of the time of harvest and storage conditions, occurring in the same way for PPO but only at early harvest, since at late harvest its activity increased. CAT activity of the exocarp decreased in R and increased in CA. Phenolic compounds stood out with higher participation in the exocarp with respect to the mesocarp, being related to AOXC. The UPLC-PAD analysis showed a higher content of epicatechin-type phenolics in the exocarp, while in the mesocarp the hydroxycinnamic acid-type phenolics stood out. The transition of the fruit from early to late harvest had an impact on the decrease in phenolics of the exocarp, contrary to those of the mesocarp which increased. CA protected Hass avocado against the development of dark spots on the exocarp and mesocarp, while refrigeration of avocados for 30 d could not prevent the appearance of dark spots on the exocarp at R30d and on the mesocarp at consumption maturity (RSL30d). Further studies are needed incorporating other hydrophilic and lipophilic antioxidant molecules to obtain more information on their participation in response to oxidative stress in Hass avocados destined for markets distant from the point of production.

## Figures and Tables

**Table 1:** Main phenolic compounds determined by UPL-PDA in Hass avocado exocarp from two harvests (early and late): at harvest (initial) and at consumption maturity after refrigeration and controlled atmosphere storage.

Peak N°	Retention Time (min)	Phenolic Compound Assignment (mg/100 g DM)/ Harvest Stage		Refrigeration			Controlled Atmosphere		
			Initial (0 d)	Shelf Life—Consumption Maturity (RSL) *			Shelf Life—Consumption Maturity (CASL) *		
				10 d	20 d	30 d	15 d	30 d	50 d
1	11.06	Chlorogenic Acid							
		Early (I)	312.9 ± 54.9 ^abA^	393.3 ± 94.0 ^abA^	442.6 ± 109.5 ^aA^	117.5 ± 18.3 ^cB^	239.1 ± 15.6 ^bcA^	107.1 ± 6.8 ^cA^	217.7 ± 69.3 ^bcA^
		Late (II)	288.3 ± 0.4 ^aA^	53.5 ± 6.1 ^eB^	61.4 ± 11.8 ^eB^	139.2 ± 19.6 ^cA^	104.9 ± 11.8 ^cdB^	118.5 ± 63.9 ^bcA^	218.0 ± 57.8 ^bA^
2	12.87	Epicatechin derivative 1 (EpiD1)							
		Early (I)	666.2 ± 100.1 ^cA^	1515.4 ± 483.5 ^abA^	2023.4 ± 352.6 ^aA^	139.8 ± 6.2 ^deB^	743.0 ± 218.1 ^cA^	1356.6 ± 555.6 ^abA^	324.0 ± 109.1 ^dA^
		Late (II)	119.3 ± 9.4 ^dB^	54.2 ± 27.6 ^eB^	167.2 ± 1.7 ^cB^	345.1 ± 74.3 ^abA^	250.9 ± 53.2 ^bB^	356.4 ± 29.6 ^aB^	249.7 ± 4.8 ^bA^
3	14.08	Epicatechin							
		Early (I)	428.9 ± 115.6 ^cdA^	1086.6 ± 229.1 ^abA^	1502.7 ± 213.6 ^aA^	298.7 ± 33.3 ^eA^	571.9 ± 134.6 ^cA^	693.8 ± 147.4 ^cA^	238.4 ± 53.6 ^eA^
		Late (II)	132.9 ± 13.2 ^bB^	72.8 ± 14.8 ^cB^	184.1 ± 25.0 ^abB^	229.0 ± 4.8 ^aB^	228.6 ± 21.9 ^aB^	205.2 ± 32.8 ^aB^	136.4 ± 13.3 ^bB^
4	16.03	Epicatechin derivative 2 (EpiD2)							
		Early (I)	429.2 ± 134.0 ^abA^	684.5 ± 121.1 ^abA^	171.5 ± 85.4 ^cA^	124.4 ± 4.5 ^cdB^	522.6 ± 147.2.6 ^abA^	792.1 ± 229.5 ^aA^	208.1 ± 42.6 ^cA^
		Late (II)	127.7 ± 20.6 ^cB^	39.8 ± 0.4 ^dB^	108.0 ± 18.8 ^cA^	197.6 ± 10.0 ^aA^	171.7 ± 19.4 ^abB^	206.7 ± 17.4 ^aB^	183.2 ± 17.7 ^aA^
5	17.16	Epicatechin derivative 3 (EpiD3)							
		Early (I)	402.5 ± 3.97 ^bc^	459.9 ± 119.0 ^b^	846.85 ± 85.6 ^a^	133.1 ± 8.5 ^d^	367.8 ± 158.4 ^bc^	484.6 ± 200.3 ^b^	141.5 ± 25.0 ^d^
		Late (II)	ND	ND	ND	ND	ND	ND	ND
6	18.20	Epicatechin derivative 4 (EpiD4)							
		Early (I)	45.3 ± 16.7 ^d^	310.7 ± 124.5 ^bc^	546.5 ± 37.5 ^a^	198.3 ± 28.8 ^c^	258.3 ± 12.4 ^bc^	420.8 ± 10.9 ^b^	153.1 ± 32.8 ^c^
		Late (II)	ND	ND	ND	ND	ND	ND	ND
7	20.07	Epicatechin derivative 5 (EpiD5)							
		Early (I)	135.9 ± 45.6 ^b^	492.3 ± 129.9 ^a^	513.6 ± 96.4 ^a^	107.8 ± 24.6 ^bc^	78.1 ± 5.1 ^c^	393.8 ± 42.9 ^ab^	183.5 ± 13.0 ^b^
		Late (II)	ND	ND	ND	ND	ND	ND	ND
Total phenolic compounds (TPC-UPLC)									
Early (I)			2421.1 ± 429.8 ^bA^	4942.9 ± 1301.4 ^abA^	6047.3 ± 541.5 ^aA^	1163.1 ± 85.8 ^cA^	2781.2 ± 691.7 ^bA^	4239.0 ± 1193.7 ^abA^	1466.5 ± 280.1 ^cA^
Late (II)			668.2 ± 42.7 ^bB^	220.3 ± 35.9 ^dB^	520.6 ± 30.3 ^cB^	911.0 ± 108.7 ^aAB^	756.2 ± 62.5 ^abB^	886.8 ± 143.7 ^abB^	787.2 ± 48.7 ^abB^

Values of each row are the mean of ten independent determinations (ten avocados) ± SD (n = 10). Different lowercase superscript letters in the same row indicate significant differences. Letters in different uppercase superscripts in the same column per compound indicate significant differences (*p* < 0.05) by the Tukey test. ND = Not detected. (*) Correspond to ready-to-eat fruit (consumption maturity) subjected to ripening in shelf life conditions after 10, 20 and 30 days of refrigeration storage or 15, 30 and 50 days of controlled atmosphere storage.

**Table 2:** Main phenolic compounds determined by UPL-PDA in Hass avocado mesocarp from two harvests (early and late): at harvest (initial) and at consumption maturity after refrigeration and controlled atmosphere storage.

Peak N°	Retention Time (min)	Phenolic Compound Assignment (mg/100 DM)/ Harvest Stage		Refrigeration			Controlled Atmosphere		
			Initial (0 d)	Shelf Life—Consumption Maturity (RSL) *			Shelf Life—Consumption Maturity (CASL) *		
				10 d	20 d	30 d	15 d	30 d	50 d
1	7.9	Syringique acid derivative (SAD)							
		Early (I)	Tr	2.92 ± 1.31 ^abA^	3.23 ± 1.34 ^abA^	3.01 ± 1.29 ^abA^	ND	2.02 ± 0.53 ^bA^	4.07 ± 1.32 ^aA^
		Late (II)	Tr	1.61 ± 0.32 ^bA^	0.31 ± 0.09 ^cB^	0.15 ± 0.01 ^cB^	ND	1.67 ± 0.39 ^bA^	3.19 ± 0.68 ^aA^
2	11.52	*p*-coumaric acid derivative 1 (PCAD1)							
		Early (I)	Tr	0.33 ± 0.16 ^dB^	0.80 ± 0.27 ^cB^	2.85 ± 1.00 ^aA^	0.65 ± 0.10 ^cB^	0.78 ± 0.31 ^cB^	1.40 ± 0.19 ^abAB^
		Late (II)	Tr	7.47 ± 2.40 ^aA^	5.34 ± 0.25 ^bA^	3.36 ± 0.48 ^cA^	6.85 ± 0.43 ^aA^	3.87 ± 0.56 ^cA^	2.14 ± 0.72 ^dA^
3	12.52	*p*-coumaric acid derivative 2 (PCAD2)							
		Early (I)	ND	ND	ND	ND	0.36 ± 0.14 ^aB^	0.27 ± 0.05 ^aB^	0.28 ± 0.02 ^aB^
		Late (II)	ND	1.40 ± 0.46 ^ab^	0.52 ± 0.12 ^b^	0.11 ± 0.02 ^c^	1.60 ± 0.22 ^aA^	0.92 ± 0.39 ^bA^	0.89 ± 0.25 ^bA^
4	13.12	Caffeic acid derivative 1 (CAD1)							
		Early (I)	ND	ND	ND	ND	ND	ND	ND
		Late (II)	ND	1.59 ± 0.85 ^ab^	2.60 ± 0.70 ^a^	ND	0.10 ± 0.03 ^c^	0.11 ± 0.03 ^c^	ND
5	13.91	*p*-coumaric acid derivative 3 (PCAD3)							
		Early (I)	ND	ND	ND	ND	ND	ND	ND
		Late (II)	ND	0.69 ± 0.03 ^a^	0.61 ± 0.10 ^a^	0.08 ± 0.02	0.07 ± 0.02 ^c^	0.15 ± 0.01 ^b^	0.06 ± 0.01 ^c^
6	16.35	*p*-coumaric acid							
		Early (I)	Tr	0.26 ± 0.01 ^bB^	ND	ND	0.50 ± 0.20 ^aB^	0.42 ± 0.09 ^aB^	0.28 ± 0.09 ^bA^
		Late (II)	Tr	1.76 ± 0.17 ^abA^	1.10 ± 0.04 ^c^	0.22 ± 0.07 ^d^	2.10 ± 0.32 ^aA^	1.80 ± 0.13 ^abA^	0.17 ± 0.01 ^dB^
7	17.18	*p*-coumaric acid derivative 4 (PCAD4)							
		Early (I)	ND	ND	ND	ND	ND	ND	ND
		Late (II)	ND	2.11 ± 0.01 ^a^	0.99 ± 0.09 ^b^	0.11 ± 0.01 ^d^	2.24 ± 0.24 ^a^	1.21 ± 0.52 ^b^	0.52 ± 0.18 ^c^
8	18.8	*p*-coumaric acid derivative 5 (PCAD5)							
		Early (I)	Tr	0.63 ± 0.06 ^cB^	1.67 ± 0.31 ^bB^	1.15 ± 0.30 ^bB^	3.93 ± 0.16 ^aB^	3.22 ± 0.86 ^aB^	3.22 ± 1.13 ^aAB^
		Late (II)	Tr	30.29 ± 7.00 ^aA^	11.44 ± 2.63 ^cA^	5.17 ± 0.94 ^dA^	23.56 ± 1.16 ^abA^	13.06 ± 5.32 ^cA^	5.18 ± 1.53 ^dA^
9	19.79	*p*-coumaric acid derivative 6 (PCAD6)							
		Early (I)	Tr	2.01 ± 0.68 ^aB^	1.96 ± 0.55 ^abA^	0.76 ± 0.28 ^cA^	2.43 ± 1.10 ^aB^	1.63 ± 0.36 ^abB^	2.43 ± 0.27 ^aA^
		Late (II)	Tr	6.89 ± 1.46 ^aA^	3.21 ± 2.16 ^bcAB^	0.32 ± 0.02 ^dB^	6.42 ± 0.27 ^aA^	4.07 ± 1.61 ^bA^	2.73 ± 0.^15 bA^
10	19.88	Caffeic acid derivative 2 (CAD2)							
		Early (I)	ND	ND	ND	ND	ND	ND	ND
		Late (II)	ND	2.33 ± 0.23 ^a^	1.70 ± 0.21 ^b^	0.17 ± 0.04 ^c^	0.70 ± 0.02 ^c^	0.85 ± 0.49 ^c^	0.17 ± 0.01 ^c^
11	20.02	*p*-coumaric acid derivative 7 (PCAD7)							
		Early (I)	ND	ND	ND	ND	ND	ND	ND
		Late (II)	ND	4.79 ± 1.47 ^a^	6.28 ± 4.69 ^a^	0.10 ± 0.01 ^d^	2.30 ± 0.11 ^ab^	0.73 ± 0.29 ^c^	0.21 ± 0.02 ^d^
12	20.54	*p*-coumaric acid derivative 8 (PCAD8)							
		Early (I)	ND	ND	ND	ND	0.17 ± 0.11 ^aB^	0.15 ± 0.04 ^aA^	0.12 ± 0.03 ^aA^
		Late (II)	ND	0.86 ± 0.20 ^a^	0.81 ± 0.63 ^a^	0.10 ± 0.03 ^b^	0.51 ± 0.10 ^aA^	0.15 ± 0.05 ^bA^	0.04 ± 0.00 ^cB^
13	20.44	*p*-coumaric acid derivative 9 (PCAD9)							
		Early (I)	ND	ND	ND	ND	0.70 ± 0.15 ^aB^	0.23 ± 0.02 ^bB^	0.24 ± 0.02 ^bB^
		Late (II)	ND	2.44 ± 0.15 ^b^	1.90 ± 1.36 ^c^	0.29 ± 0.31 ^d^	5.51 ± 0.53 ^aA^	3.10 ± 1.5 ^bA^	2.20 ± 0.75 ^bcA^
Total phenolic compounds (TPC-UPLC)									
Early (I)			ND	6.15 ± 2.21 ^bB^	7.66 ± 2.46 ^bB^	7.76 ± 0.29 ^bB^	8.74 ± 1.96 ^abB^	8.73 ± 2.27 ^abB^	10.18 ± 0.33 ^aB^
Late (II)			ND	64.22 ± 14.21 ^aA^	36.83 ± 11.48 ^cA^	10.17 ± 0.05 ^eA^	51.96 ± 1.06 ^abA^	31.71 ± 12.29 ^cA^	17.50 ± 4.20 ^dA^

Values of each row are the mean of ten independent determinations (ten avocados) ± SD (n = 10). Different lowercase superscript letters in the same row indicate significant differences. Letters in different uppercase superscripts in the same column per compound indicate significant differences (*p* < 0.05) by the Tukey test. Tr = Traces. ND = Not detected. (*) Correspond to ready-to-eat fruit (edible ripeness) subjected to ripening at shelf life conditions after 10, 20 and 30 days of refrigeration storage or 15, 30 and 50 days of controlled atmosphere storage.

## Data Availability

All data are shown in the Figures and Supplementary Figures.

## Supplementary Materials

The following supporting information can be downloaded at: https://www.mdpi.com/article/10.3390/plants12234008/s1, Figure S1: qualitative–quantitative scale to qualify the appearance of dark spots at exocarp level (a) and pulp browning (b) and vascular browning (c) at mesocarp level; Figure S2: biplot displaying the samples and variables overlaid for the whole dataset: early and middle harvests, at harvest and ready to eat or consumption maturity (SL).
