# Supplementary material for: Postharvest Storage Differentially Modulates the Enzymatic and Non-Enzymatic Antioxidant System of the Exocarp and Mesocarp of Hass Avocado: Implications for Disorders
Source: Plants (Basel). 2023 Nov 29;12(23):4008. doi: 10.3390/plants12234008 (PMC10707783; doi:10.3390/plants12234008)
Supplement: Supplementary file 1 [file plants-12-04008-s001.zip › plants-2701311-supplementary.pdf]

Supplementary Figures

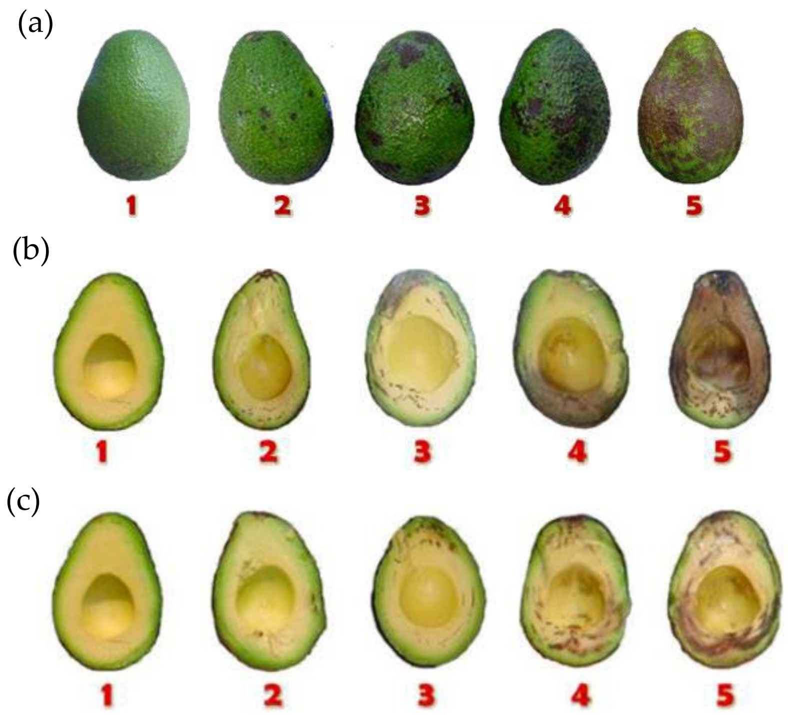

Figure S1. Qualitative-quantitative scale to qualify the appearance of dark spots at exocarp level (a) and pulp browning (b) and vascular browning (c) at mesocarp level.

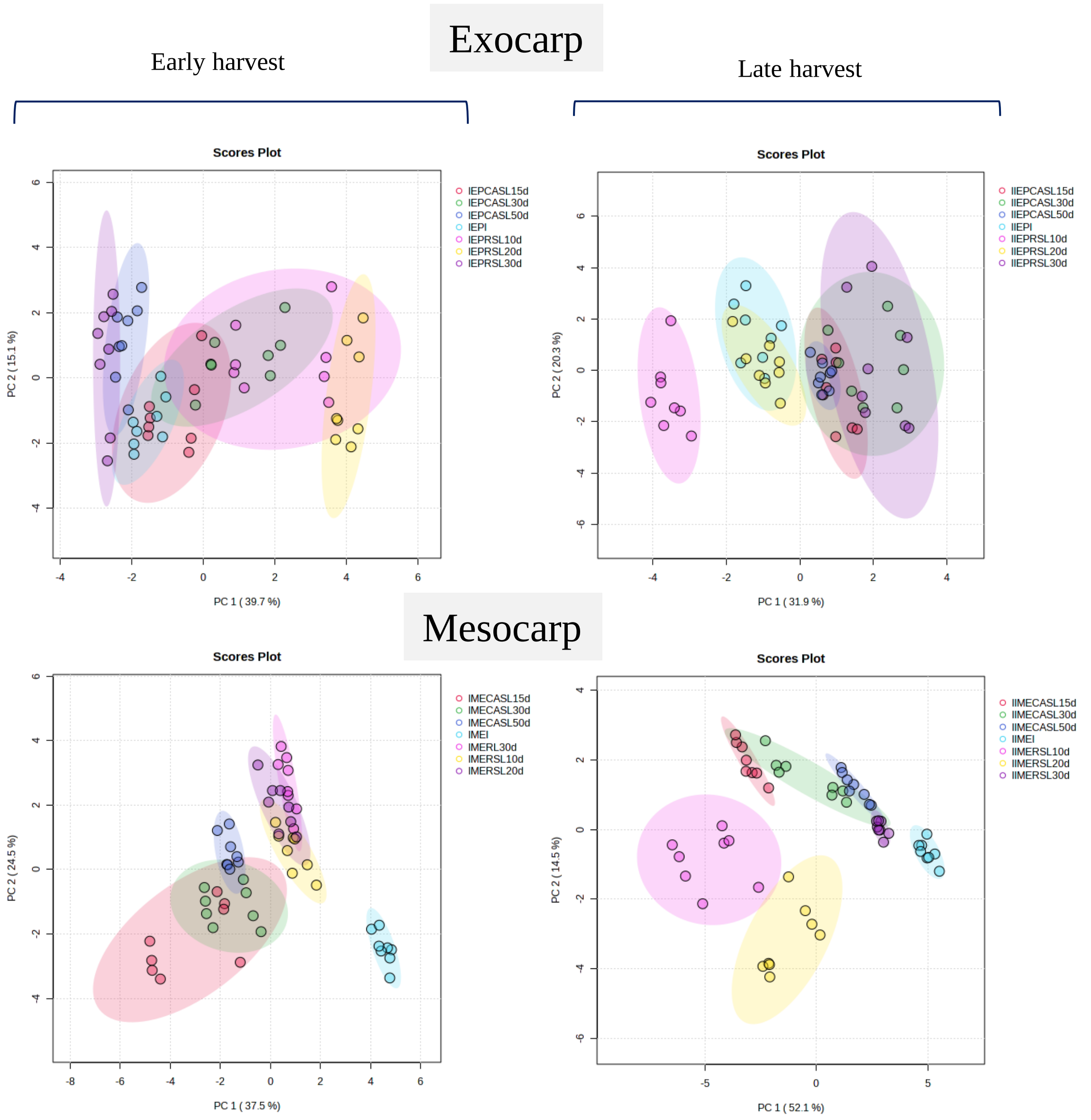

Figure S2. Biplot displaying the samples and variables overlaid for the whole dataset: early and middle harvest fruit, at harvest and ready to eat. IMEI and IIMEI = initial samples at early (I) and late (II) harvests. R10d, R20d and R30d = avocados cold stored for 10, 20 and 30 d RSL10d, RSL20d and RSL30d = ready to eat avocadoes from cold storage at 10, 20 and 30 d. CA15d, CA30d and CA50d = avocados stored for 15, 30 and 50 d under controlled atmosohere. CASL15d, CASL30d and CASL30d = avocados at the ready to eat stage from CA storage for 10, 20 and 30 d.
